# Integrated biocontrol of tobacco bacterial wilt by antagonistic bacteria and marigold

**DOI:** 10.1038/s41598-021-95741-w

**Published:** 2021-08-11

**Authors:** Yun Hu, Wan Zhao, Xihong Li, Ji Feng, Chunli Li, Xiaoqiong Yang, Qingqing Guo, Lin Wang, Shouwen Chen, Yanyan Li, Yong Yang

**Affiliations:** 1 State Key Laboratory of Biocatalysis and Enzyme Engineering, School of Life Science, Hubei University, Wuhan, 430062 China; 2 Tobacco Research Institute of Hubei Province, Wuhan, 430030 China; 3 Hubei Tobacco Industry Co., Ltd., Wuhan, 430040 China

**Keywords:** Applied microbiology, Microbial communities

## Abstract

Tobacco bacterial wilt (TBW) is seriously damages the growth of tobacco. There is an urgent need to find a safer and more effective measure to control TBW. In this study, *B. amyloliquefaciens* ZM9 and marigold powder were applied to the tobacco roots alone or in combination, and the potential inhibition of TBW was assessed. On the other hand, the effects of these treatments on soil physicochemical properties, rhizosphere microbial community and soil metabolites were also evaluated. The results showed that the application of *B. amyloliquefaciens* ZM9 or marigold powder alone significantly reduced the abundance of *R. solanacearum* in rhizosphere soil, while the integrated treatment showed the strongest inhibitory effect. Moreover, the integrated treatment can inhibit the secretion of chemoattractants, and affect the change of rhizosphere soil microbial composition. In conclusion, the combination of antagonistic bacteria agent *B. amyloliquefaciens* ZM9 with marigold powder can enhance the suppression of TBW. Furthermore, *B. amyloliquefaciens* ZM9 and marigold have synergistic effects on suppressing TBW by regulation soil physicochemical properties, soil metabolites and microbial structure. This study provide a promising strategy for TBW control by integrated applying of *B. amyloliquefaciens* ZM9 and marigold powder.

## Introduction

Bacterial wilt is a typical soil-borne disease caused by *R. solanacearum*, which seriously damages the growth of tobacco^1^. It is distributed in almost all the flue-cured tobacco growing areas in the world, especially in tropical and subtropical tobacco areas, which is a major devastating disease threatening world tobacco production and causing great economy loss^2^. Therefore, the control of bacterial wilt has been a worldwide problem. Lots of researches on the control of bacterial wilt in breeding resistant varieties, chemical control, agricultural control. However, these traditional control methods have limited efficacy and many problems, such as lack of resistant varieties, poor control efficacy, pathogen resistant, environmental pollution, and so on^3,4^. There is an urgent need to find effective and environmentally friendly measures to control bacterial wilt.

Biocontrol is widely used for prevention and control of bacterial wilt^5^. Antagonistic bacteria, as the most common method of biocontrol can reduce the harm of pathogens to plants, and promote plant growth^6,7^. The main biocontrol bacterial include *Bacillus* spp., *Pseudomonas* spp., *Streptomyces* spp. and so on^8–10^. Among them, the bioactive peptides produced by *Bacillus* spp. have potential inhibitory effect on plant pathogens, and bacillus can form spore state under condition stress, which is easy to be stored and transported as stable products^11,12^. Our previous study showed that *B. amyloliquefaciens* ZM9 as an efficient biocontrol agent can suppress tobacco bacterial wilt (TBW)^13,14^. However, the control effect of single antagonistic bacteria in different regions and different crops is very different, and it is easy to be affected by external climate factors and farming conditions, resulting in unstable control^15^.

Marigold (*Tagetes erecta* L.) is a common garden flower, which can be used for ornamental, medical and pharmaceutical purposes^16^. It is reported that marigold contains allelopathy compounds (such as α-terthiophene) and has antibacterial activity, which can effectively protect plants against parasitic nematodes^17,18^. Marigold also has been reported to successfully suppress *R. solanacearum* when used as a rotational or intercropping plant under greenhouse conditions^19^. In our previous study, we found that marigold and tobacco intercropping can effectively reduce the incidence of TBW, affect the soil microbial community and soil physicochemical properties^20^. Kamal et al. found that the combination of biocontrol agents and resistance inducers could make biocontrol agents better adapt to environment changes and effectively control of bacterial wilt^21^. Thus, integrated control approaches should be taken into consideration for controlling bacterial wilt more effectively.

In this study, a field trail was carried out in a TBW prone area in Enshi City of Hubei province, China. Antagonistic bacteria *B. amyloliquefaciens* ZM9 and antibacterial plant marigold were applied to tobacco field. The main objectives of this study were to (1) evaluate the potential of antagonistic bacteria *B. amyloliquefaciens* ZM9 and antibacterial plant marigold, alone or in combination, to suppress TBW; and (2) investigate the effects of these treatments on soil physicochemical properties, rhizosphere microbial community and soil metabolites. We hypothesized that application of *B. amyloliquefaciens* ZM9 and marigold could alter soil physicochemical properties, microbial abundance and soil metabolites, thus inhibiting TBW. This study proposed a new approach to control TBW by combining of *B. amyloliquefaciens* ZM9 and marigold.

## Results

### The incidence and index of TBW

Symptoms of TBW were recorded at 50 days, 70 days and 90 days post-transplantation, and the disease incidence (I) and disease index (DI) of TBW were calculated. From 50 to 90 days post-transplantation, the I and DI gradually increased. In addition, the I and DI of the CK group were significantly higher than other treatment groups. While, the I and DI of T1 group were significantly lower than other treatment groups (Table 1). The results indicated that T1, T2 and T3 groups can effectively restrained the incidence and severity of TBW, and the T1 treatment group had the best inhibitory effect among all treatment groups.

**Table 1:** The occurrence of tobacco bacterial wilt of four treatments.

Treatments	50 days post-transplantation		70 days post-transplantation		90 days post-transplantation	
	Disease incidence (%)	Disease index	Disease incidence (%)	Disease index	Disease incidence (%)	Disease index
CK	26.37 ± 0.59 a	19.23 ± 1.12 a	56.56 ± 2.98 a	37.37 ± 1.18 a	92.00 ± 3.56 a	47.52 ± 1.25 a
T1	5.06 ± 2.18 c	3.42 ± 0.48 c	6.95 ± 1.24 c	5.02 ± 0.25 c	17.33 ± 2.54 c	8.28 ± 0.60 c
T2	7.28 ± 1.05 bc	6.85 ± 0.23 b	20.36 ± 2.51 b	12.97 ± 1.93 b	24.33 ± 1.85 b	15.89 ± 0.18 b
T3	8.98 ± 1.11 b	7.67 ± 1.17 b	21.13 ± 1.59 b	13.47 ± 1.92 b	25.07 ± 2.31 b	16.45 ± 1.90 b

All data are presented as the mean ± SE. CK: the control group, T1: marigold powder mix with *B. amyloliquefaciens* ZM9 group, T2: *B. amyloliquefaciens* ZM9 group, T3: marigold powder. The different letters in the same column indicate significant differences at *p* < 0.05 according to LSD test.

### Rhizosphere soil physicochemical properties

Four physicochemical properties of the rhizosphere soil at 0 days, 50 days, 70 days and 90 days post-transplantation were analyzed (Table 2). At 0 day, there was no significant difference in pH, hydrolysable nitrogen (HN), available phosphorous (AP) and available potassium (AK) among different treatment groups. From 50 to 90 days post-transplantation, there was significant difference in pH, HN, AP and AK between the CK group and other treatment groups. The HN content in the CK group was observed higher than that in other treatment groups, and gradually increased from 50 to 90 days post-transplantation. While, among all treatment groups, the T1 treatment group contained the lowest HN. Additionally, the values of pH, AP and AK were lowest in the CK group and highest in T1 treatment group. The AP and AK content in the T1 treatment group increased from 50 to 70 days post-transplantation, and decreased at 90 d post-transplantation (Table 2). What’s more, pH (Pearson =  − 0.843, *p* = 0.031), AP (Pearson =  − 0.286, *p* = 0.002) and AK (Pearson =  − 0.725, *p* = 0.005) showed significantly negative correlation with the incidence of TBW (Table S1).

**Table 2:** Soil chemical properties in four treatments at 0 days, 50 days, 70 days and 90 days post-transplanted, respectively.

	HN (mg/kg)	AP (mg/kg)	AK (mg/kg)	pH
CK_0d	108.52 ± 12.16 a	35.33 ± 0.44 a	267.97 ± 9.77 a	6.24 ± 0.05 a
T1_0d	110.49 ± 9.73 a	35.51 ± 0.86 a	267.96 ± 8.62 a	6.25 ± 0.03 a
T2_0d	109.17 ± 5.24 a	35.43 ± 1.13 a	272.05 ± 6.55 a	6.23 ± 0.05 a
T3_0d	108.91 ± 6.56 a	35.91 ± 1.24 a	271.50 ± 9.76 a	6.25 ± 0.03 a
CK_50d	154.07 ± 10.76 a	40.46 ± 2.02 c	386.91 ± 3.77 d	5.35 ± 0.02 c
T1_50d	121.75 ± 1.38 b	64.08 ± 3.65 a	1085.53 ± 2.53 a	5.98 ± 0.03 a
T2_50d	123.32 ± 2.46 b	49.12 ± 1.79 b	915.72 ± 5.42 b	5.53 ± 0.09 c
T3_50d	133.43 ± 2.27 b	49.59 ± 2.13 b	754.54 ± 12.31 c	5.74 ± 0.12 b
CK_70d	175.69 ± 3.09 a	51.14 ± 2.74 c	849.22 ± 12.44 d	4.53 ± 0.01 c
T1_70d	145.92 ± 4.39 c	80.63 ± 1.65 a	1596.84 ± 1.84 a	5.28 ± 0.06 a
T2_70d	160.17 ± 0.85 b	71.47 ± 3.99 b	1344.11 ± 6.41 b	5.12 ± 0.03 b
T3_70d	163.26 ± 2.43 b	72.17 ± 3.82 b	1057.53 ± 16.38 c	5.11 ± 0.04 b
CK_90d	167.43 ± 4.14 a	44.72 ± 1.87 c	682.46 ± 11.21 d	5.03 ± 0.03 c
T1_90d	131.53 ± 2.22 b	76.23 ± 3.31 a	1335.63 ± 4.08 a	5.64 ± 0.03 a
T2_90d	135.00 ± 3.00 b	63.70 ± 1.41 b	929.20 ± 1.10 b	5.18 ± 0.06 b
T3_90d	133.93 ± 2.40 b	63.75 ± 2.71 b	890.65 ± 12.62 c	5.20 ± 0.03 b

Soil chemical properties in soils are presented as the mean ± SE. CK: the control group, T1: *B. amyloliquefaciens* ZM9 mix with marigold powder group, T2: *B. amyloliquefaciens* ZM9 group, T3: marigold powder. The different letters in the same column indicate significant differences as determined by LSD test. *p* < 0.05.

### Bacterial diversity and community structure in soil

In total, 163,609 high-quality raw sequences with an average length of 251 bps for bacteria were obtained from rhizosphere soil samples after removing low-quality reads. The OTUs, Chao1 and Shannon index were used to evaluate and compare the diversity and richness of bacterial community among different treatment groups (Table S2). From 50 to 90 days post-transplantation, the OTUs and Shannon index were both significantly lower in the CK group than in the other treatment groups. Analysis by Chao1, a lower richness of bacteria was also found in the CK group. Moreover, the OTUs, Chao1 and Shannon index were higher in the T1 treatment group than in the other treatment groups. These results indicated that T1 treatment group had a higher bacteria diversity and richness than the other treatment groups. This suggested that T1 treatment group could effectively improve bacterial community diversity.

**Figure 1:**
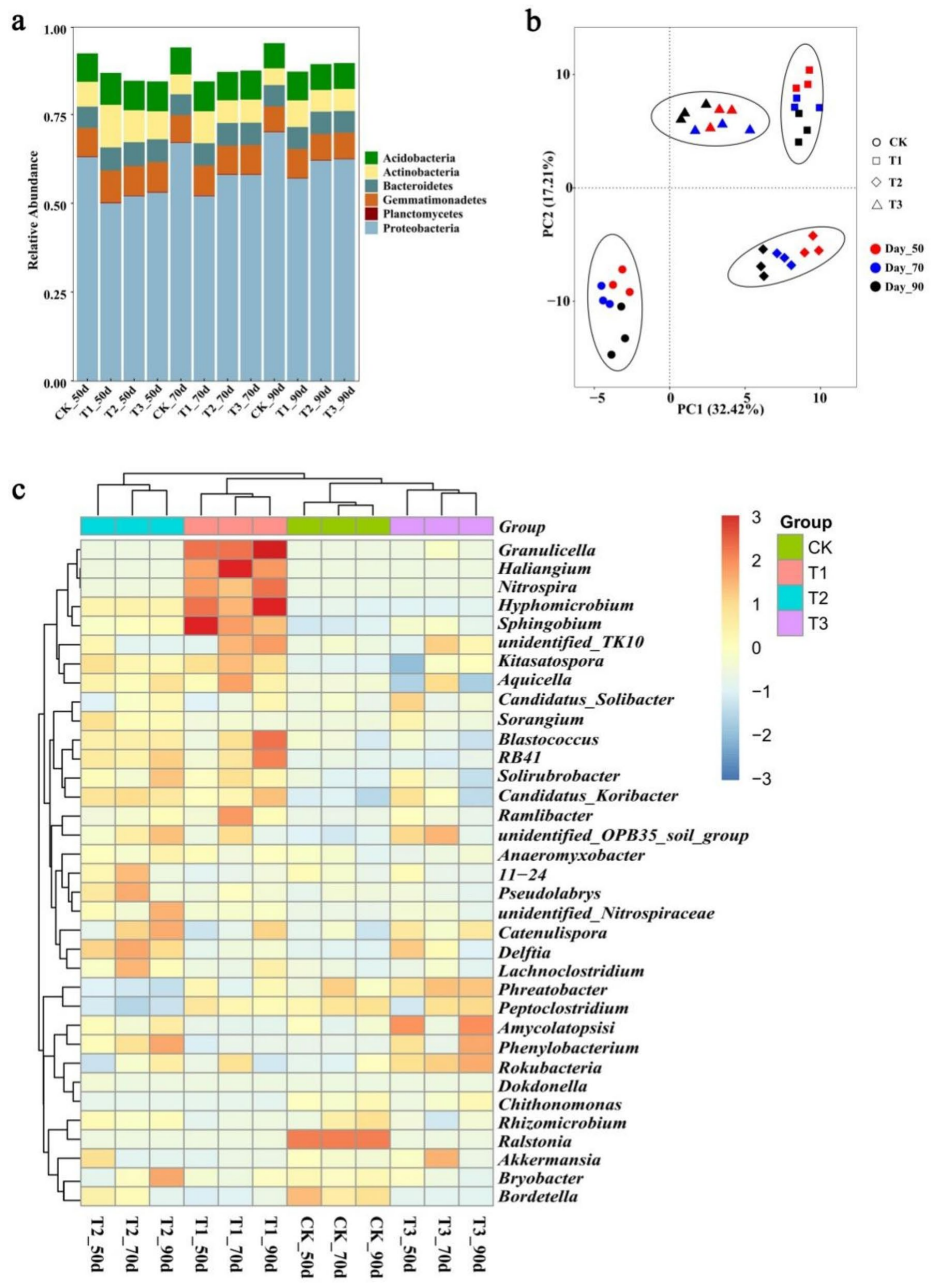
Soil bacterial community in four treatments at 50 days, 70 days and 90 days post-transplanted, respectively. **(a)** The relative abundance of bacterial phyla in soil samples. **(b)** The Principal components analyzed (PCA) of soil bacterial community. **(c)** Hierarchical cluster analysis of predominant bacterial genera. (CK: the control group, T1: marigold powder mix with *B. amyloliquefaciens* ZM9 group, T2: *B. amyloliquefaciens* ZM9 group, T3: marigold powder).

Bacterial were identified as 48 phyla from all soil samples. The top six abundant bacterial phyla were selected to compare the changes of bacterial communities in rhizosphere soil of different treatments (Fig. 1a). From 50 to 90 days post-transplantation, *Proteobacteria* was dominant (50.0–70.0%), followed by *Actinobacteria* (5.6–12.0%), *Acidobacteria* (7.1–9.0%), *Gemmatimonadetes* (7.1–9.0%), *Bacteroidetes* (5.9–6.7%) and *Planctomycetes* (0.20–0.2%). The relative abundance of the phylum *Proteobacteria* included the pathogen *R. solanacearum* was higher in the CK than other treatment groups. On the other hand, *Actinobacteria*, *Acidobacteria* and *Gemmatimonadetes* were abundant in T1 treatment compared to the other treatments. In addition, the relative abundance of *Proteobacteria* was increased from 50 to 90 days post-transplantation. While the relative abundance of other phyla were slight decreased from 50 to 90 days post-transplantation, indicating the relative abundance of bacterial phyla changed during different tobacco growth stages. PCA analyses based on the weighted Unifrac distance showed differences in soil bacterial community structure among different treatments. PC1 and PC2 explained 32.42% and 17.21% of the total bacterial community variations respectively. T1 (T1_50, T1_70, T1_90), T2 (T2_50, T2_70, T2_90), T3 (T3_50, T3_70, T3_90) and CK (CK_50, CK_70, CK_90) treatments were respectively clustered together and separated from each other (Fig. 1b). The results of the PCA suggested different treatments played important impact on the structure of soil bacterial community. The Heatmap analysis of the top 35 genera with hierarchical clusters was used to identify the different composition of bacterial community structure (Fig. 1c). Different treatments in different period were divided into four broad categories, suggesting there were distinction of bacterial community structure in different treatments. Additionally, *Ralstonia* were significant higher in CK treatment than the other treatments. While *Granulicella*, *Hyphomicrobium*, *Haliangium*, *Nitrospira*, *Sphingoblum* were more abundant in the T1 treatment than the other treatments.

### Fungal diversity and community structure in soil

**Figure 2:**
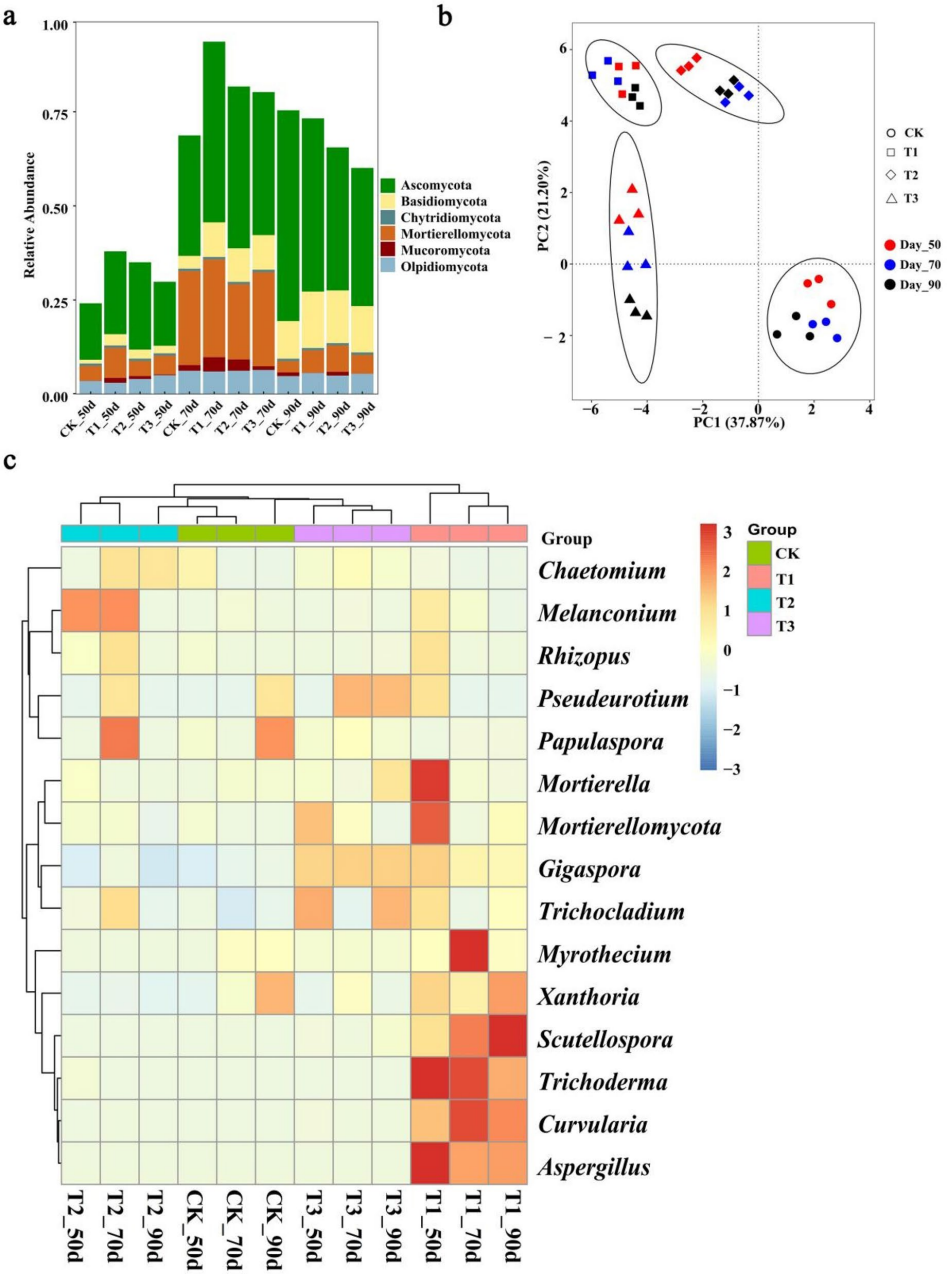
Soil fungal community in four treatments at 50 d, 70d and 90 d post-transplanted, respectively. **(a)** The relative abundance of fungal phyla in soil samples. **(b)** Hierarchical cluster analysis of predominant fungal genera. **(c)** The Principal components analyzed (PCA) of soil fungal community. (CK: the control group, T1: marigold powder mix with *B. amyloliquefaciens* ZM9 group, T2: *B. amyloliquefaciens* ZM9 group, T3: marigold powder).

All rhizosphere soil samples consist of 31,663 high-quality raw sequences for fungal. The difference of the OTUs, Chao1 and Shannon index of fungal community among treatment groups were also analyzed (Table S2). The OTUs, Chao1 and Shannon index were also higher in the T1 treatment group than in the other treatment groups during all stages of tobacco growth. Thereby, the T1 treatment group has a higher fungal diversity and richness than the other treatment groups. Six main known fungal phyla were identified from all soil samples, including *Ascomycota* (15.0–56.0%), followed by *Mortierellomycota* (4.0–26.0%), *Basidiomycota* (1.0–12.3%), *Olpidiomycota* (3.0–6.4%), *Mucoromycota* (0–3.8%) and *Chytridiomycota* (0.6%) (Fig. 2a). Overall, the total relative abundance of six fungal phyla was low at 50 days, and increased at 70 days, while different fungal phyla varied irregularly, suggesting fungal phyla changed during different tobacco growth stages. According to PCA analysis, PC1 and PC2 explained 59.07% of the total fungal community (Fig. 2b). The fungal community of different treatments was separated from each other, and different tobacco growth stages within one treatment showed close distances, indicating different treatments had influence on fungal community. In the Heatmap for fungal community structure (Fig. 2c), four broad categories were divided, and the antagonistic fungal (such as *Curvularia*, *Trichoderma*, *Scutellospora*, *Aspergillus*) were more abundant in the T1 treatment than the other treatments.

### The relative abundance of *R. Solanacearum*

**Figure 3:**
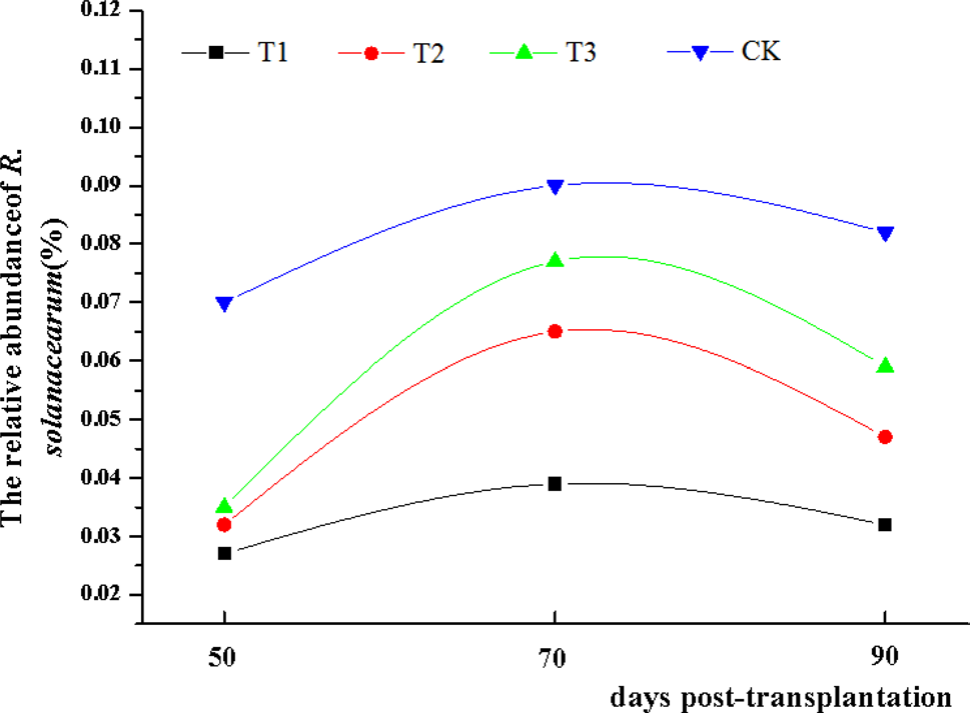
The relative abundance of *R. solanacearum* in rhizosphere soil from four treatments at 50 days, 70 days and 90 days post-transplanted, respectively. CK: the control group, T1: marigold powder mix with *B. amyloliquefaciens* ZM9 group, T2: *B. amyloliquefaciens* ZM9 group, T3: marigold powder.

The relative abundance of *R. solanacearum* in the rhizosphere soil of different treatment groups at different tobacco growth periods were analyzed. The variation trend of the relative abundance of *R. solanacearum* in different treatment groups was similar. The relative abundance of *R. solanacearum* increased from 50 to 70 days and decreased from 70 to 90 days. From 50 to 90 days post-transplantation, the relative abundance of *R. solanacearum* in the CK was significantly higher than other treatment groups. Additionally, the relative abundance of *R. solanacearum* in the T1 group was the lowest among all treatment groups (Fig. 3).

### Soil metabolites from different treatments

**Figure 4:**
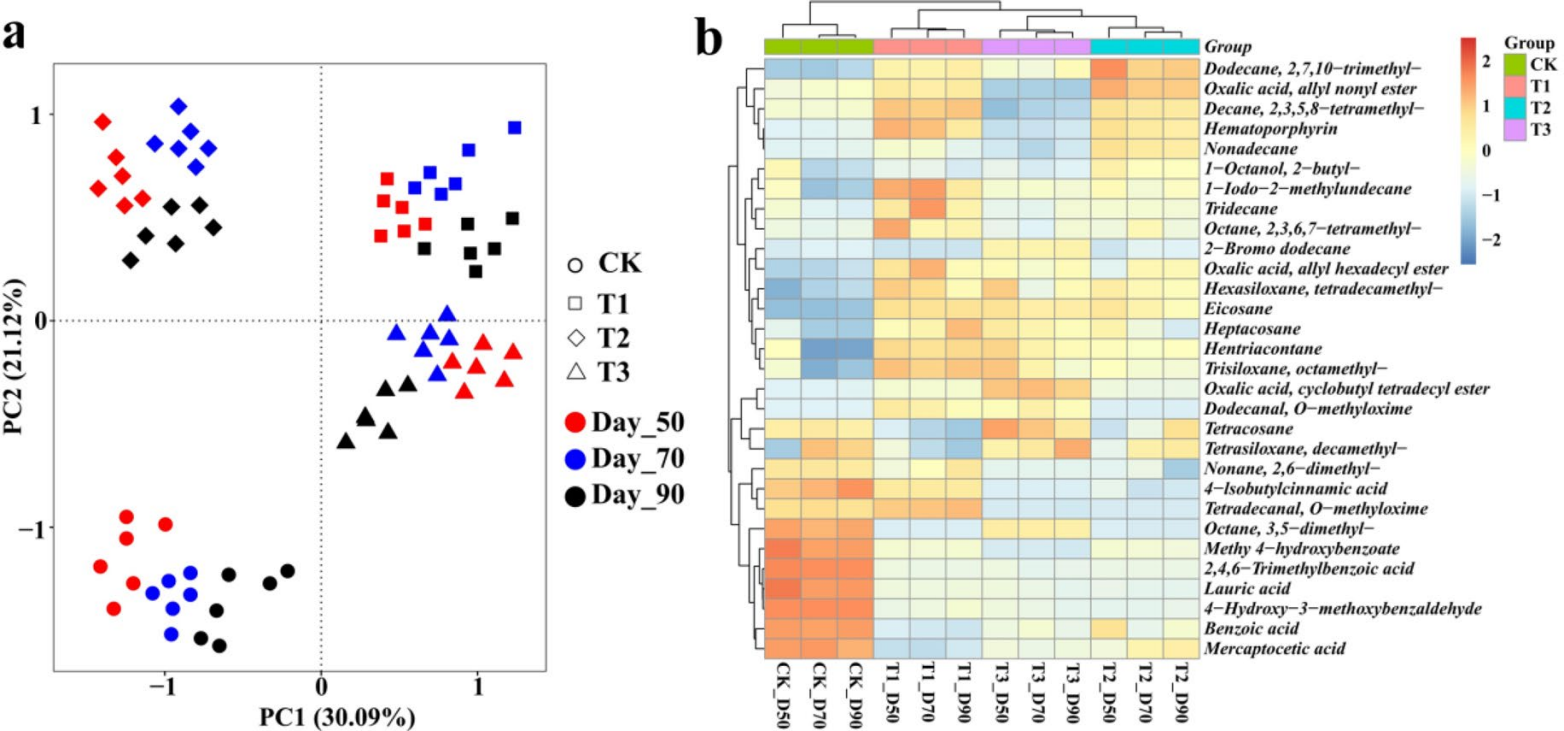
**(a)** Principal component analysis (PCA) of metabolites analyzed from the four treatments soil at 50 days, 70 days and 90 days post-transplanted, respectively. **(b)** Heatmap showing top 30 most significant metabolites from soil samples of four treatments at 50 days, 70 days and 90 days post-transplanted. (CK: the control group, T1: marigold powder mix with *B. amyloliquefaciens* ZM9 group, T2: *B. amyloliquefaciens* ZM9 group, T3: marigold powder).

GC-TOF–MS analysis of 80% (v/v) methanol crude extracts resulted in identification of 46 features were identified in all soil samples. PCA was applied to understand the clustering features of different treatments soil metabolites at different tobacco growth periods. The first components (PC1) showed 30.09% difference in variation, and PC2 explained 21.12% of the variance (Fig. 4a). Different treatments were respectively clustered together and separated from each other, indicating different treatments play impact on soil metabolites. The Heatmap detailed the soil metabolites concentration of different treatments based on the top 30 significant metabolites (Fig. 4b). The concentration of benzoic acid, lauric acid, 4-hydroxy-3-methoxybenzaldehyde, methyl 4-hydroxybenzoate and mercaptoacetic acid were obviously higher in the CK than those in the other treatments.

### Relationships among microbial community, soil physicochemical properties and metabolites

**Figure 5:**
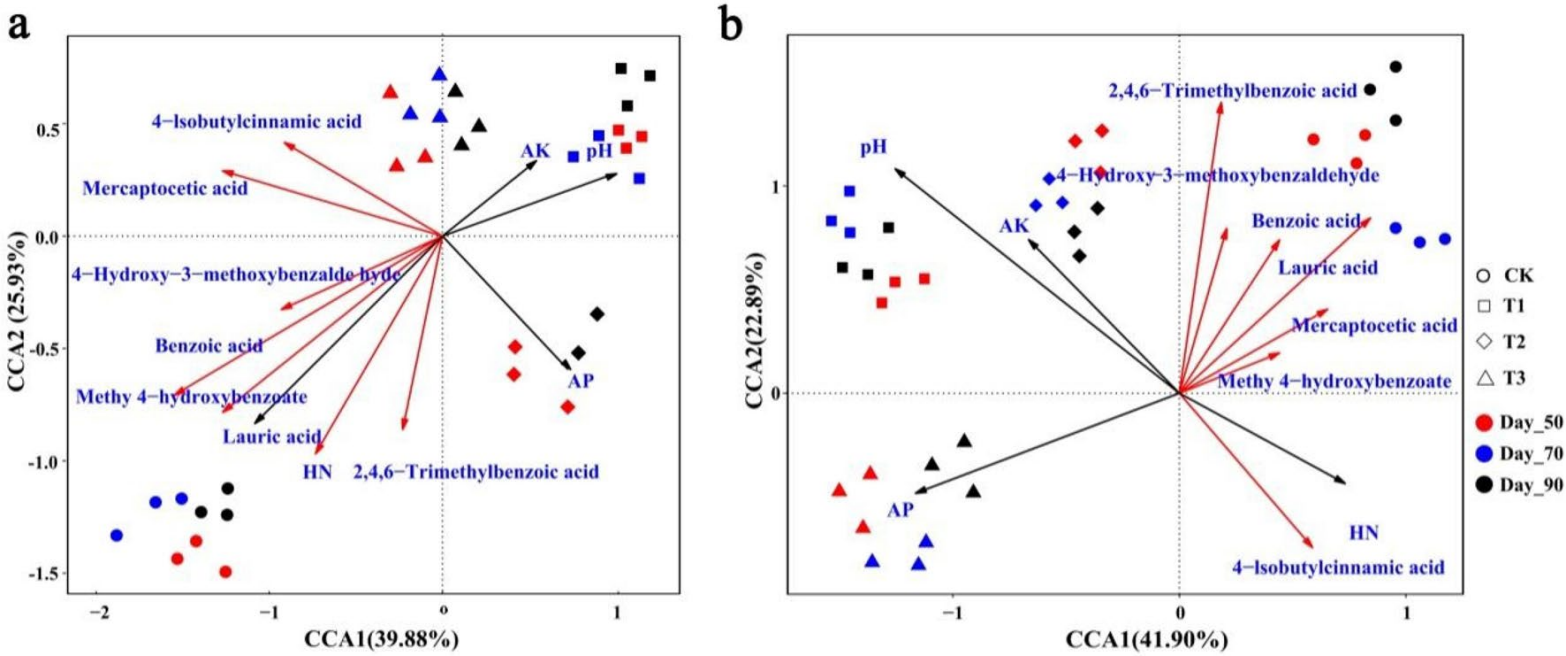
Canonical correspondence analysis (CCA) of the relative abundance of microbial community with soil physicochemical properties and metabolites. **(a)** Soil bacterial community, **(b)** soil fungal community. The soil properties and metabolites are indicated with arrows, including soil pH, hydrolysable nitrogen (HN), available phosphorous (AP), available potassium (AK), benzoic acid, lauric acid, 4-hydroxy-3-methoxybenzaldehyde, methyl 4-hydroxybenzoate, 4-Isobutylcinnamic acid, 2,4,6-trimethylbenzoic acid and mercaptoacetic acid. CK: the control group, T1: marigold powder mix with *B. amyloliquefaciens* ZM9 group, T2: *B. amyloliquefaciens* ZM9 group, T3: marigold powder. The percentage of variation is explained by each axis.

Canonical correspondence analysis (CCA) was used to investigate the relationships among microbial community, soil physicochemical properties and metabolites. Eleven factors including pH, HN, AP, AK, benzoic acid, lauric acid, 4-hydroxy-3-methoxybenzaldehyde, methyl 4-hydroxybenzoate, 4-Isobutylcinnamic acid, 2,4,6-trimethylbenzoic acid and mercaptoacetic acid were selected for CCA. The results showed that the treatments of T1, T2, T3 and CK were separated from each other (Fig. 5). These variables explained 65.81% and 64.79% of bacterial and fungal community variation, respectively. The bacterial community composition in the T1 group were positively correlated with pH, AK and AP, but negatively correlated with HN, 4-hydroxy-3-methoxybenzaldehyde, benzoic acid, methyl 4-hydroxybenzoate, lauric acid, 2,4,6-trimethylbenzoic acid, 4-isobutylcinnamic acid and mercaptoacetic acid. While the bacterial community composition in the CK group were positively correlated with HN, 4-hydroxy-3-methoxybenzaldehyde, benzoic acid, methyl 4-hydroxybenzoate, lauric acid, 2,4,6-trimethylbenzoic acid and mercaptoacetic acid (Fig. 5a). For fungi, the fist canonical axis was positively correlated with 2,4,6-trimethylbenzoic acid, 4-hydroxy-3-methoxybenzaldehyde, lauric acid, benzoic acid, mercaptoacetic acid and methyl 4-hydroxybenzoate, the second axis was positively correlated with pH and AK (Fig. 5b). As important variables, pH, AK, AP, HN, benzoic acid, lauric acid, mercaptoacetic acid and 4-hydroxy-3-methoxybenzaldehyde played major roles in the shaping of soil microbial community.

## Discussion

In our previous study, *B. amyoliquefaciens* ZM9 as an efficient biocontrol agent can reduce TBW by producing lipopeptides to against *R. solanacearum* and changing the tobacco rhizosphere microbial community^13^. Marigold is reported to have antibacterial activity, and effectively reduce the incidence of TBW^18,20^. The application of single biocontrol agent is difficult to perform sustainable and efficient against pathogens of the plant under diverse soil environmental conditions. Hence, antibacterial plant assistance of antagonistic bacteria should be considered as an effective integrated biocontrol method to improve the colonization ability of antagonistic bacteria and control bacterial wilt. In order to achieve more sustainable and efficient control, we applied *B. amyloliquefaciens* ZM9 and marigold powder to tobacco fields in different ways. We have demonstrated that *B. amyloliquefaciens* ZM9 and marigold power had potential for controlling bacterial wilt. Furthermore, the combination of *B. amyloliquefaciens* ZM9 and marigold power could more effective suppress TBW than single application of *B. amyloliquefaciens* ZM9 or marigold. The integrated biocontrol method significantly suppressed TBW through changing soil chemical properties, rhizosphere microbial community and soil metabolites.

Hooks et al. (2010) summaried that the mechanism of marigold inhibiting nematodes includes enhancing the activity of antagonistic microorganisms^17^. Our study also found that marigold powder could promote antagonistic bacteria *B. amyloliquefaciens* ZM9 (Fig S1). Terblanche and De Villiers (1998) showed that marigold not only reduced the pathogen population in the soil, but also prevented the tobacco plants from developing symptoms^22^. In our present study, the application of marigold powder reduced the TBW incidence and index (Table 1), which was consistent with previous study^22^. In addtion, the combination of *B. amyloliquefaciens* ZM9 and marigold powder significantly reduced the disease incidence and index compared with single application of *B. amyloliquefaciens* ZM9 or marigold powder (Table 1). This consistent with some researches which indicated that the application of biocontrol agents alone is unlikely to perform consistently on plant pathogens in different rhizosphere soil environments. While, the integrated biocontrol method is more effective in reducing the disease incidence and index of bacterial wilt, and this could be attributed to different mechanisms in disease suppression or competition for nutrients^21,23–25^.

Several studies have demonstrated that increasing pH is important for inhibiting the survival of *R. Solanacearum*, and soil pH as an environmental factor can regulate the soil physicochemical properties, metabolites and microbial community composition^20,26–28^. Nutrient P has also been reported to meet the need of plant growth and increase the soil microbial diversity, and the increased P supply significantly decreased the relative density of *R. Solanacearum*^29,30^. In this study, rhizosphere soil properties pH, AP and AK were negatively correlated with the incidence of TBW. Additionally, the T1 (*B. amyloliquefaciens* ZM9 mix with marigold powder group) had a higher pH value, AP and AK content than other treatments, consistently with previous report^29^. We infer that the integrated application of *B. amyloliquefaciens* ZM9 and marigold powder may reduce the incidence of TBW by changing soil physicochemical properties.

In the current study, the OTUs, Chao1 and Shannon index of soil microbial community were higher in the T1 treatment than in the other treatments, indicating the integrated application of *B. amyloliquefaciens* ZM9 and marigold powder could improve the richness and diversity of microbial community. In addition, the soil microbial community varies at different tobacco growth stages. The results of PCA, suggesting different treatments and growth stages could influence soil microbial community structure, which was similar to previous report^20,31^. It was also testified by the Heatmap, showing the difference of microbial community structure in different treatments. Moreover, *Granulicella*, *Hyphomicrobium*, *Haliangium*, *Nitrospira*, *Sphingoblum*, *Curvularia*, *Trichoderma*, *Scutellospora*, *Aspergillus* were more abundant in the T1 treatment. Previously reports showed that most of these genera have beneficial effects on soil nutrient cycling, the genera *Hyphomicrobium* and *Nitrospira* played important role in nitrogen cycling^32,33^. *Granulicella* was found to use different substances as carbon sources and participate in carbon cycling in the soil^34^. Besides, *Haliangium* and *Sphingoblum* was reported as beneficial rhizosphere microorganisms can mitigate many soil-borne diseases and assist plant growth by increasing plant health and growth^35,36^. The antagonistic microorganisms *Trichoderm*a, *Scutellospora* and *Aspergillus* were reported to interact directly with roots to produce bioactive substances that promote plant growth and resist biotic and abiotic stress^37,38^. In accordance with other researches, the inhibition of bacterial wilt could not be attributed to a single bacterial or fungal group, and this inhibition is most likely governed by microbial consortia networks^39,40^. Results also showed that the relative abundance of *R. solanacearum* in the T1 groups was the lowest among all treatment groups. Therefore, the integrated application of *B. amyloliquefaciens* ZM9 and marigold powder can affect soil microbial community and rebuild healthy soil microbial community composition to against tobacco plants from pathogen infection.

Soil metabolomics could provide insight into the complex interactions of plants, soil and microorganisms^41–43^. According to the previous investigation, the occurrence of soil-borne disease is closely related with root exudates^44^. Some studies have also found that organic acids benzoic acid and lauric acid from tobacco root exudates can simulate the growth of *R. solanacearum*^45,46^. Phenolic acid 4-hydroxy-3-methoxybenzaldehyde as allelochemical with high concentration can inhibit the growth of plant and promote the occurrence of soil-born disease^47,48^. Hasegawa et al. (2019) showed several aromatic acid secreted by plants are chemoattractants of *R. Solanacearum*^49^*.* In our study, metabolites benzoic acid, lauric acid, 4-hydroxy-3-methoxybenzaldehyde and mercaptoacetic acid were found notably higher in the CK group and lower in T1 treatment, consistent with the previous findings^45–48^. On the other hand, Terblanche and De Villiers (1998) demonstrated that the secondary metabolites of marigold could inhibit *R. Solanacearum*. Therefore, the integrated application of *B. amyloliquefaciens* ZM9 and marigold powder may reduce the incidence of bacterial wilt by regulating root exudates^22^. Metabolomic analysis of the soil studied indicated a separation among different treatment during different tobacco growth stages. The content of soil metabolites increased with the growth of tobacco, which was probably due to the accumulation of root exudates. Furthermore, the content of root exudates increased at different tobacco growth stages, which had a great impact on soil microbial community.

After CCA, the relationships among microbial community, soil physicochemical properties and metabolites showed that pH, AK, AP, HN, benzoic acid, lauric acid, mercaptoacetic acid and 4-hydroxy-3-methoxybenzaldehyde played major roles in the shaping of soil microbial community. In accordance with other studies, soil physicochemical properties related to the microbial community structure, and high pH and AP has been linked to a higher abundance of microbial^31^. Besides, some metabolites were also correlated with microbial abundance^50^. Therefore the changes of soil microbial community at different tobacco growth stages was possibly due to the changes of soil metabolites and soil physicochemical properties^31,49^.

## Conclusions

Application of antagonistic bacteria *B. amyloliquefaciens* ZM9 or antibacterial plant marigold powder alone showed effective biocontrol of TBW. The integrated application of a combination of *B. amyloliquefaciens* ZM9 and marigold powder showed more effectiveness in suppressing TBW than application of either *B. amyloliquefaciens* ZM9 or marigold power alone. *B. amyloliquefaciens* ZM9 and marigold have synergistic effects suppressed TBW by regulation soil physicochemical properties, microbial structure and soil metabolites. Our results provide a promising strategy for TBW control by integrated applying of *B. amyloliquefaciens* ZM9 and marigold powder.

## Materials and methods

### Field experiment

The field experiment was carried out in a continuous cropping tobacco field in Xuan’en County (109.26° E, 29.59° N), Enshi City, Hubei province, China from April to September 2019. The field experimental was carried out according to the rules of flue-cured tobacco cultivation (GB/T 23221-2008). The field, 480 m^2^ in size, and the incidence of TBW was higher than 95% every year for the past five years. Tobacco seeds of Yunyan87 were provided by the Tobacco Research Institute of Hubei, Wuhan, China. The seeds were grown in floating polystyrene trays in a greenhouse for approximately 60 days before being transplanted to the field. 5 g compound fertilizer, 10 g potassium phosphate, 450 g ash soil and 50 g tobacco straw fertilizer were applied to each tobacco plant when transplantation^13^. *B. amyloliquefaciens* ZM9 (Genbank: KF906355.1) was used as the antagonistic bacteria in this study. The roots and stems of marigold discarded by local farmers were collected, air-dried, smashed and filtered for 20 mesh as marigold powder. The experimental design consisted of three blocks, each 160 m^2^ in size, each block was divided into four plots of 40 m^2^, 60 plants in each plots. Four treatments: (1) the control group (CK, without any pesticide); (2) *B. amyloliquefaciens* ZM9 mix with marigold powder group (T1): *B. amyloliquefaciens* ZM9 was incubated as previous study^51^, 1 g of marigold powder was mixed with the 100 mL of *B. amyloliquefaciens* ZM9 culture (1.0 × 10^7^ CFU/mL) and then irrigated into the tobacco roots when transplantation. The effect of marigold powder on *B. amyloliquefaciens* ZM9 was evaluated by the plate count method^13^ (Fig. S1); (3) *B. amyloliquefaciens* ZM9 group (T2): 100 mL of *B. amyloliquefaciens* ZM9 culture (1.0 × 10^7^ CFU/mL) irrigated into the tobacco roots when transplantation; and (4) marigold powder group (T3): 1 g of marigold powder was applied to the tobacco roots when transplantation. The planting density of all treatments were the same, and all treatments and replicates were randomly placed in the field.

### Rhizosphere soil sampling and physicochemical properties analysis

Rhizosphere soil were collected by five-spot-sampling method at 0 days, 50 days, 70 days and 90 days post-transplantation when recording the disease occurrence. Then the soil samples from the five separate sites were mixed to one soil sample, and partitioned into two subsamples, one stored at − 80 °C for microbiological and metabolome analysis, and another for physicochemical properties analysis after air-dry. The analysis of soil pH, hydrolysable nitrogen (HN), available phosphorous (AP) and available potassium (AK) was performed according to previous study^52^.

### Disease incidence and index

At 50 d, 70 d and 90 d post-transplantation, the symptoms of TBW were monitored at five different sites. The TBW disease index (DI) based on severity scale of 0–9 was described in a previous studies^14,53^. Briefly, “0” represents the plants without visible symptoms; “1” represents the presence of occasional chlorotic spots on stems, or less than half of the leaves wilted on unilateral stems; “3” represents the presence of a black streak less than half the height of the stem, or between half to two-thirds of the leaves wilted on unilateral stems; “5” represents the presence of a black streak over half the length of the stem, but not reaching the top of the stem, or more than two-thirds of the leaves wilted on unilateral stems; “7” represents the presence of a black streak reaching the top of the stem, or all leaves wilted; and “9” represents the dead plant. Based on the number of plants in each rating scale, disease incidence (I) and disease index (DI) of TBW were calculated as I = n′/ N × 100% and DI = ∑(r × n)/(N × 9) × 100, where n′ is the total number of infected tobacco plants, r is the rating scale of disease severity, n is the number of infected tobacco plants with a rating of r, and N is the total number of plants.

### Soil DNA extraction

Soil DNA was extracted from 0.5 g rhizosphere soil using the FastDNA Spin Kit (MP Biomedicals, USA), following the manufacture’s protocol. The integrity of DNA samples were determinde by 1% agarose gel electrophoresis. Then the concentration and purity of the DNA were determined using a Nanodrop ND-1000 Spectrophotmeter (Nanodrop Techenologies, USA)^14^.

### DNA sequence data collection and analysis

The extracted soil genomic DNA was used as template to amplify 16S rRNA and ITS rRNA genes according to our previous study^14^. The V4 regions of 16S rRNA gene were amplified using primers 515F (5’ GTGCCAGCMGCCGCGGTAA 3’) and 806R (5’ GGACTACHVGGGTWTCTAAT 3’)^13^, while the ITS1 regions of ITS rRNA gene were amplified using primers ITS5-1737F (5′-GGAAGTAAAAGTCGTAACAAGG-3′) and ITS2-2043R (5′-GCTGCGTTCTTCATCGATGC-3′)^26^. The library was sequenced on an Illumina HiSeq platform (Novogene Bioinformatics Technology Co., Ltd, China). The sequence quality was statistically analyzed by CASAVA1.8. The raw sequence data was preliminarily filtrate using the FASTX Toolkit 0.0.13 software package, removing the low mass base at the tail of the sequence (Q value less than 20) and the sequences with lengths less than 35 bp. Finally, the length of the valid reads was approximately 250 bp. All effective tags of all samples were clustered using Uparse software (V7.0.1001, http://drive5.com/uparse/). Sequences with ≥ 99.5% identity for 16S rDNA and sequences with ≥ 97% identity for ITS were assigned to the same OTUs (operational taxonomic units). The OTUs, Chao1 and Shannon index were calculated with QIIME (Version 1.7.0) to evaluate richness and diversity of soil microbial community. The principal components analyzed (PCA) with the weighted Unifrac distance was carried out using software R (R Core Team 2014).

### Soil metabolite extraction

The ground soil (1 g) was extracted in 5 mL 80% (v/v) methanol (10 min, 20 °C) using sonicator. The residue was reextracted twice with the same procedure and the total combined supernatant was filtered through Whatman filter paper (125 mm). The supernatant was dried in a vacuum concentrator without heating. The extracts obtained were stored at − 80 °C for GC-TOF–MS analysis^54^.

### GC-TOF–MS analysis and data preprocessing

The dried extracts were added to 60 μL of a methoxyamination hydrochloride in pyridine (20 mg mL^−1^), and incubated at 80 ℃ for 30 min, for methoxymation. Then, 80 μL of the BSTFA regent (1% TMCS, v/v) was added, and resulting solutions were incubated at 70 ℃ for 1.5 h. All samples were analyzed by gas chromatograph system coupled with a Pegasus HT time-of-flight mass spectrometer (GC-TOF–MS)^55^.

GC-TOF–MS analysis was performed using an Agilent 7890 gas chromatograph system (Agilent Technologies Inc., USA) coupled with a Pegasus HT time-of-flight mass spectrometer (LECO Corporation, USA). The system utilized a DB-5MS capillary column (30 m, i.d. 250 μm, and film thickness 0.25 μm; Agilent Technologies Inc., USA), was employed, applying positive electron ionization (70 eV). Full-scan mode were acquired over the 50–500 Da mass (scan rate of 12.5 scans per second) applying a solvent delay of 4.78 min. The injection, transfer line, and ion source temperatures were 280, 280, and 250 °C, respectively. The samples (1 μL) were injected in splitless mode. Helium was used as the carrier gas, the front inlet purge flow was 3 mL min^−1^, and the gas flow rate through the column was 1 mL min^−1^. The initial temperature of the oven was 50 °C for 1 min, raised at a rate of 20 °C min^−1^ to 310 °C, then kept for 6 min at 310°C^55^.

Chroma TOF 4.3X software (LECO Corporation, USA) and the LECO-Fiehn Rtx5 database were used for raw peaks exacting, data baselines filtering and calibration, peak alignment, deconvolution analysis, peak identification and integration of the peak area. Both of mass spectrum match and retention index match were considered in metabolites identification.

### Statistical analysis

Differences between treatment groups were assessed by one-way analysis of variance (ANOVA) and least significant difference (LSD) test (p < 0.05). Correlation analysis between disease incidence of TBW and soil physicochemical properties was conducted by Pearson (2-tailed). Heatmaps analyses based on soil microbial community were generated using software R (R Core Team 2014). Canonical correspondence analysis (CCA) used software R (R Core Team 2014) to analyze the relationships among microbial community structure, soil physicochemical properties and metabolites.

## Supplementary Information

Supplementary Information.
