# Supplementary material for: Integrated biocontrol of tobacco bacterial wilt by antagonistic bacteria and marigold
Source: Sci Rep. 2021 Aug 11;11:16360. doi: 10.1038/s41598-021-95741-w (PMC8357815; doi:10.1038/s41598-021-95741-w)
Supplement: Supplementary file 1 — Supplementary Information. [file 41598_2021_95741_MOESM1_ESM.docx]

Supporting information

**Integrated biocontrol of tobacco bacterial wilt by antagonistic bacteria and marigold**

**Yun Hu^1a^, Wan Zhao^1a^, Xihong Li^2^, Ji Feng^2^, Chunli Li^1^, Xiaoqiong Yang^1^, Qingqing Guo^1^, Lin Wang^3^, Shouwen Chen^1^, Yanyan Li^2*^ & Yong Yang^1*^**

^1^State Key Laboratory of Biocatalysis and Enzyme Engineering, School of life science, Hubei University, Wuhan 430062, China.

^2^Tobacco Research Institute of Hubei Province, Wuhan 430030, China.

^3^Hubei Tobacco Industry Co., Ltd., Wuhan 430040, China.

*E-mails: [yangyong@hubu.edu.cn,](mailto:yangyong@hubu.edu.cn,) [yanyanli0025@126.com](mailto:yanyanli0025@126.com).

^a^Authors contributed equally to this work.

**
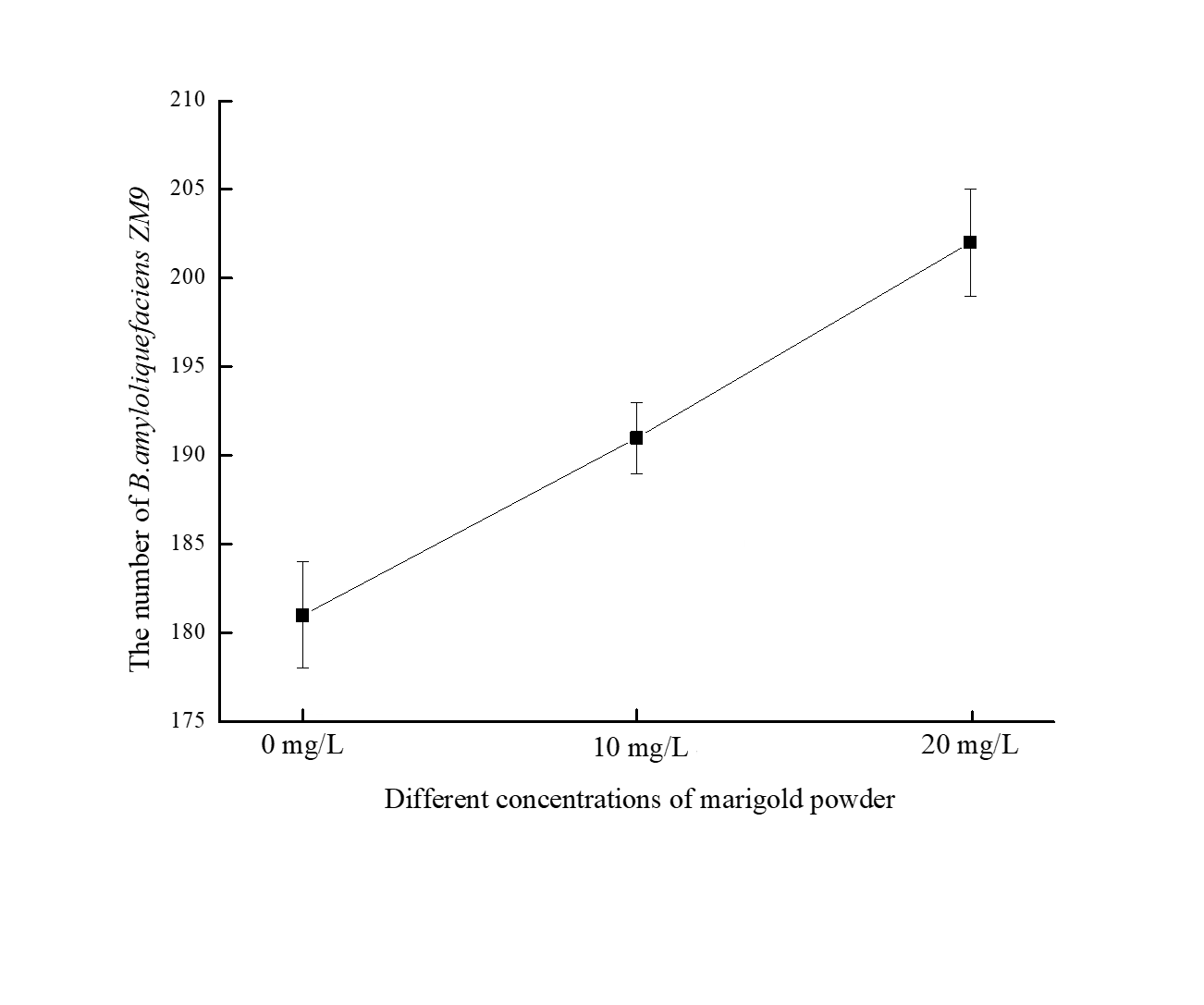
**

**Figure S1.** Effect of marigold powder on *B. amyloliquefaciens* ZM9

**Table S1** Pearson correlation analysis between disease incidence of tobacco bacterial wilt and soil physicochemical properties.

|  | **HN** | **AP** | **AK** | **pH** |
| --- | --- | --- | --- | --- |
| **Pearson** | 0.907** | − 0.286** | − 0.725** | −0.843* |
| ***p* value** | 0.003 | 0.002 | 0.005 | 0.031 |

* and ** indicate correlation is significant at *p* < 0.05 and *p* < 0.01

**Table S2** Alpha diversity index of bacterial and fungal of four treatments at 50 d, 70 d and 90 d post-transplanted, respectively.

| Treatment | Bacterial | | | Fungal | | |
| --- | --- | --- | --- | --- | --- | --- |
|  | OTUs | Chao1 | Shannon | OTUs | Chao1 | Shannon |
| CK_50d | 4788 ± 354.17 b | 3493 ± 217.89 c | 8.34 ± 0.63 b | 422 ± 37.87 c | 544 ± 33.58 c | 4.68 ± 0.70 b |
| T1_50d | 6209 ± 195.58 a | 4249 ± 177.58 a | 9.48 ± 0.38 a | 572 ± 50.64 a | 800 ± 88.46 a | 5.19 ± 0.44 a |
| T2_50d | 6098 ± 264.78 a | 3900 ± 256.84 b | 9.20 ± 0.11 a | 545 ± 46.87 b | 756 ± 80.23 b | 5.14 ± 0.21 a |
| T3_50d | 5783 ± 213.47 a | 3858 ± 174.31 b | 9.31 ± 0.08 a | 533 ± 42.44 b | 743 ± 19.76 b | 5.05 ± 0.78 a |
| CK_70d | 4133 ± 317.46 c | 3149 ± 653.73 c | 7.97 ± 0.41 b | 530 ± 17.89 c | 622 ± 64.77 b | 4.32 ± 0.15 c |
| T1_70d | 5321 ± 244.45 a | 4084 ± 579.94 a | 8.99 ± 0.67 a | 868 ± 62.55 a | 908 ± 73.56 a | 6.43 ± 0.88 a |
| T2_70d | 5167 ± 374.23 a | 3820 ± 452.75 b | 8.56 ± 0.33 ab | 741 ± 51.73 b | 876 ± 29.86 a | 5.98 ± 0.43 b |
| T3_70d | 4890 ± 517.97 b | 3785 ± 417.74 b | 8.6 ± 1.03 b | 744 ± 77.72 b | 869 ± 69.44 a | 6.21 ± 0.74 ab |
| CK_90d | 4891 ± 347.65 b | 3674 ± 334.31 c | 8.07 ± 0.62 c | 611 ± 80.52 b | 643 ± 65.31 b | 4.43 ± 0.45 c |
| T1_90d | 5689 ± 408.89 a | 4591 ± 174.31 a | 9.21 ± 0.14 a | 697 ± 30.15 a | 786 ± 58.70 a | 5.99 ± 0.21 a |
| T2_90d | 5329 ± 496.67 a | 4251 ± 80.53 bc | 8.63 ± 0.21 b | 634 ± 41.27 b | 745 ± 67.44 a | 5.63 ± 0.62 b |
| T3_90d | 5054 ± 291.35 b | 4182 ± 326.98 b | 8.69 ± 0.87 b | 627 ± 21.98 b | 723 ± 60.11 ab | 5.67 ± 0.84 b |

CK: the control group, T1: marigold powder mix with *B. amyloliquefaciens* ZM9 group, T2: *B. amyloliquefaciens* ZM9 group, T3: marigold powder. The different letters in the same column indicate significant differences at p < 0.05 according to LSD test.
